# Intramuscular Neural Distribution of Rhomboid Muscles: Evaluation for Botulinum Toxin Injection Using Modified Sihler’s Method

**DOI:** 10.3390/toxins12050289

**Published:** 2020-05-03

**Authors:** Kyu-Ho Yi, Hyung-Jin Lee, You-Jin Choi, Ji-Hyun Lee, Kyung-Seok Hu, Hee-Jin Kim

**Affiliations:** 1 Inje County Public Health Center, Inje 24633, Korea; kyuho90@daum.net; 2 Division in Anatomy and Developmental Biology, Department of Oral Biology, Human Identification Research Institute, BK21 PLUS Project, Yonsei University College of Dentistry, 50-1 Yonsei-ro, Seodaemun-gu, Seoul 03722, Korea; leehj221@yuhs.ac (H.-J.L.); cyj7797@yuhs.ac (Y.-J.C.); jh_anatomy@naver.com (J.-H.L.); hks318@yuhs.ac (K.-S.H.); 3 Department of Materials Science & Engineering, College of Engineering, Yonsei University, Seoul 03722, Korea

**Keywords:** botulinum toxin, injection, clinical guideline, rhomboid muscle, spasticity

## Abstract

This study describes the nerve entry point and intramuscular nerve branching of the rhomboid major and minor, providing essential information for improved performance of botulinum toxin injections and electromyography. A modified Sihler method was performed on the rhomboid major and minor muscles (10 specimens each). The nerve entry point and intramuscular arborization areas were identified in terms of the spinous processes and medial and lateral angles of the scapula. The nerve entry point for both the rhomboid major and minor was found in the middle muscular area between levels C7 and T1. The intramuscular neural distribution for the rhomboid minor had the largest arborization patterns in the medial and lateral sections between levels C7 and T1. The rhomboid major muscle had the largest arborization area in the middle section between levels T1 and T5. In conclusion, botulinum neurotoxin injection and electromyography should be administered in the medial and lateral sections of C7−T1 for the rhomboid minor and the middle section of T1−T7 for the rhomboid major. Injections in the middle section of C7−T1 should also be avoided to prevent mechanical injury to the nerve trunk. Clinicians can administer safe and effective treatments with botulinum toxin injections and other types of injections by following the methods in our study.

## 1. Introduction

Spasticity is a major contributor to movement disorders involving central nervous system impairment, such as stroke and brain injury [1]. Patients with hemiplegic neurologic impairment frequently suffer from shoulder spasticity, which limits shoulder movement and causes shoulder pain [2,3,4,5]. The rhomboid major and minor muscles are the core muscles targeted for treatment in patients with shoulder spasticity [4]. Involuntary activations of spastic rhomboid muscles cause the scapula to be in an elevated and medially rotated position [6]. The reduction of involuntary activations in spastic rhomboid muscles may result in proper positioning of the scapula with coordinated movement of the glenohumeral joint. Botulinum toxin (BoNT) is considered a leading therapy for the reduction of shoulder spasticity [5,7,8]. The intramuscular injection of BoNT interferes with neural transmission by decreasing the release of acetylcholine at the neuromuscular junction and deactivates muscle contraction [9].

Currently, BoNT injections are known to be among the safest and most effective methods for alleviating spasticity [10,11,12,13]. The amount of BoNT should be sufficient to allow toxin levels into the arborized area of neural distribution. The effect of the BoNT depends on uptake by the presynaptic membrane of the motor neuron at the neuromuscular junction; therefore, the injection should be given into the neuromuscular junction area [14,15,16]. The significance of using neuromuscular-junction-targeted BoNT injections has been confirmed in a clinical study on biceps brachii muscle and iliopsoas muscle. The neuromuscular-junction-targeted injection resulted in much greater volume reduction than those seen in the control groups [17,18].

However, a high dose of BoNT can cause the toxin to disperse to nearby muscles and cause undesirable paralysis [19,20]. Furthermore, frequent and excessively high doses of BoNT in injections lead to the production of antibodies that decrease the effect of treatment [19,20,21]. Therefore, to minimize the side effects and maximize the efficacy, the BoNT should be injected into the arborized zones. Many studies on the anatomical locations of arborized areas of targeted muscles have been published [22,23,24,25]. A previous study reported dorsal scapular neuropathy after injective treatment on the rhomboid muscle [26]. This was caused by an injection targeted at the entry point of the muscle, not at the arborized zones.

However, to date, no studies have revealed the intramuscular nerve distributions and arborized areas of the rhomboid muscles. In this study, we used the modified Sihler staining technique, which is a whole-mount staining technique that effectively displays intramuscular nerve distributions without damaging nerves.

The aims of this study were to elucidate the intramuscular nerve branching patterns and determine the arborized areas of the rhomboid muscles using Sihler staining. The results of this study allow the identification of effective and safe injection points for BoNT in patients with shoulder spasticity.

## 2. Results

### 2.1. Locations of the Nerve Entry Points

From the sample of dissected specimens, 28 of the 30 had a dorsal scapular nerve entry point in the middle section of C7−T1, and 2 of the 30 specimens had a dorsal scapular nerve entry point in the middle section of T1−T2.

### 2.2. Intramuscular Arborization Patterns of the Rhomboid Minor

**Figure 1:**
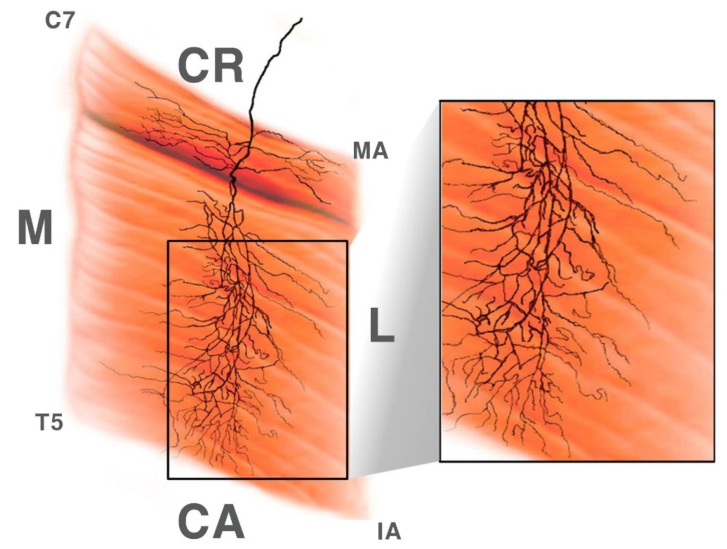
Schematic view of the right side of the rhomboid muscle and intramuscular nerve distribution. (C7: spinous process of C7, T5: spinous process of T5, MA: medial angle, IA: inferior angle, M: medial, L: lateral, CR: cranial, CA: caudal).

According to the dissection and modified Sihler staining method, 18 of the 30 rhomboid minor muscles had two regions in which the arborization patterns were the largest: the medial section of C7− T1 and the lateral section of C7−T1. Additionally, 10 had the largest arborization patterns in the medial section of C7−T1 and middle section of C7−T1. The remaining two specimens appeared to have the largest arborization patterns in the middle and lateral section of C7 to T1. A schematic image of an intramuscular arborization pattern is shown in Figure 1.

### 2.3. Intramuscular Arborization Patterns of the Rhomboid Major

The dissection and modified Sihler staining method demonstrated that a total of 26 of the 30 rhomboid major muscles had four sections in which the arborization patterns were the largest, in the middle section of T1−T5; three muscles had the largest arborization patterns in the middle section of T1−T3 and lateral section of T3−T5; one had the largest arborization patterns in the middle section of T1−T3 and medial section of T3−T5.

## 3. Discussion

Rhomboid muscles act as scapular rotators and retractors. The muscles are located beneath the middle trapezius. The dorsal scapular nerve, which originates from spinal nerve root C5, innervates the rhomboid muscles. The rhomboid muscles can be divided into the rhomboid minor and major muscles. The rhomboid minor originates from the spinous process of C7–T1 and inserts on the medial angle of the scapula. The rhomboid major muscle originates from the spinous processes of the vertebrae T2 to T5 and inserts on the medial border of the scapula [27].

Previous studies have shown the extramuscular running pattern of the dorsal scapular nerve but not intramuscular nerve innervation, which is important for treatments and diagnostic tools involving injections and electromyography [28].

There are studies that showed that periscapular muscles with focal spasticity could be managed with BoNT injections. In these clinical studies, BoNT injections were guided by electromyography and focal tenderness, not by intramuscular neural innervations. However, these treatments did show improvement in the limited range of motion and a reduction in shoulder pain [6].

The nerve entry point is where the nerve enters the muscle. The nerve entry point is targeted for the treatment of myofascial pain syndrome, involving injections of local anesthetics [29,30,31]. These treatments are known to relieve pain. However, no research regarding the rhomboid muscles as nerve entry points has been conducted.

The rhomboid muscles play an important role in needle electromyography, as they are one of the few muscles with C5 innervation only [32]. Needle EMG is a tool used for evaluating neuromuscular disorders, whereby a needle electrode is inserted into the targeted muscle [33]. Scapular winging may occur when the dorsal scapular nerve is damaged. Many studies have reported this occurrence, especially in sport players with repetitive stretching and forceful shoulder movement damage to the dorsal scapular nerve [28,33,34]. Dorsal scapular nerve damage is diagnosed by electromyography. Therefore, the intramuscular neural distribution that we provided can be used to guide the practice of needle electromyography.

Injections of BoNT are used not only for spasticity but also for atypical post-traumatic dystonia [35,36,37]. The study by Lee et al. targeted the rhomboid muscles with BoNT, and it was shown to be successful in lowering the frequency of dystonic movements [36]. Aldo et al. reported that botulinum toxin showed a significant decrease in pain and, to a lesser extent, dystonic movement in patients [35].

Injection techniques targeting the rhomboid muscles have been shown to be associated with many side effects. Lee et al. reported a patient who developed right dorsal scapular neuropathy after trigger point injection which directly damaged nerve trunk on the right rhomboid muscle [26]. They have urged clinicians to exercise caution when injecting near the medial border of the scapula. However, our findings indicate that the middle section between C7 and T1 should be avoided in order to prevent neuropathy.

Nerve damage can occasionally occur following injectable treatments targeting the muscle, due to direct needle traumas, such as EMG, trigger point injection, and BoNT injection [38,39,40]. In order to avoid any damage to the nerve, it is necessary that clinicians have knowledge of the exact neural distribution.

Furthermore, for regional anesthesia, rhomboid intercostal blocks targeting the T3−T9 levels are mainly performed by penetrating the rhomboid muscle, which may damage the dorsal scapular nerve [41,42]. Here, we suggest avoiding the middle section of the rhomboid muscles for these procedures.

Previous studies administered amounts of BoNT injection to the rhomboid muscle ranging from 10 to 50 units with one to three injection sites [6,43]. Seol et al. reported that the thickness of the rhomboid major muscle is 9–10 mm [44]. BoNT usually spreads up to 2–4 cm from the injection site [16]. The research regarding diffusion of BoNT has recommended multiple injections to prevent spread beyond the targeted muscle.

## 4. Conclusions

**Figure 2:**
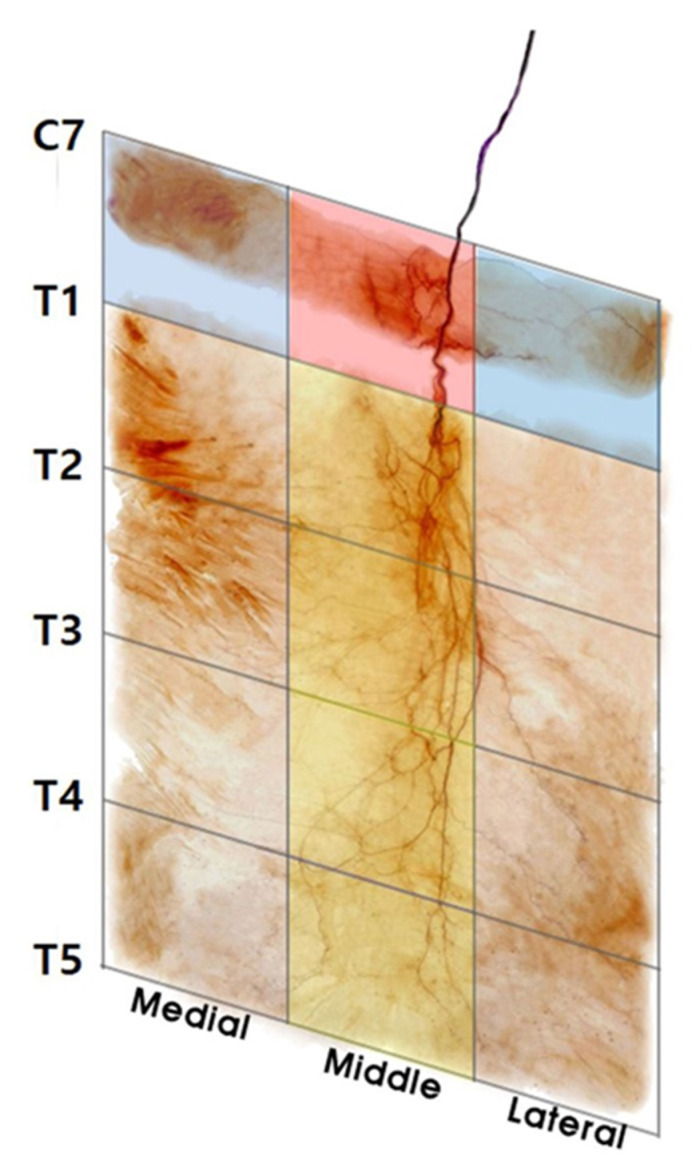
Guidelines for selective botulinum toxin A injection in the rhomboid muscles. The arborized portion of the rhomboid minor muscle was located in the medial and lateral section of C7−T1, and that of the rhomboid major was located in the middle section of T1−T5. The figure represents the right side of the rhomboid specimen.

In conclusion, we recommend that clinicians perform injections at multiple sites (six or more) with a low dose of BoNT (each 10 units) for maximum effect and to avoid side effects. We also suggest that BoNT injections and electromyography are administered in the medial and lateral sections of C7−T1 for the rhomboid minor and the middle section of T1−T7 for the rhomboid major (Figure 2). In the case of BoNT injections, the nerve entry point of the middle section of C7−T1 should be avoided to prevent damage to the nerve trunk. Clinicians can increase the effectiveness of BoNT injections and other treatments and diagnostic methods by following the methods in this study.

## 5. Materials and Methods

This study was performed in accordance with the principles outlined in the Declaration of Helsinki. Appropriate consent and approval were obtained from the families of the cadavers before the dissections were performed. A total of 30 rhomboid major and 30 minor muscles from Korean cadavers (12 men and 3 women with a mean age of 73.2 years; range, 60–96 years) were dissected, and 10 of each were subjected to modified Sihler staining to elucidate the intramuscular nerve arborization patterns.

**Figure 3:**
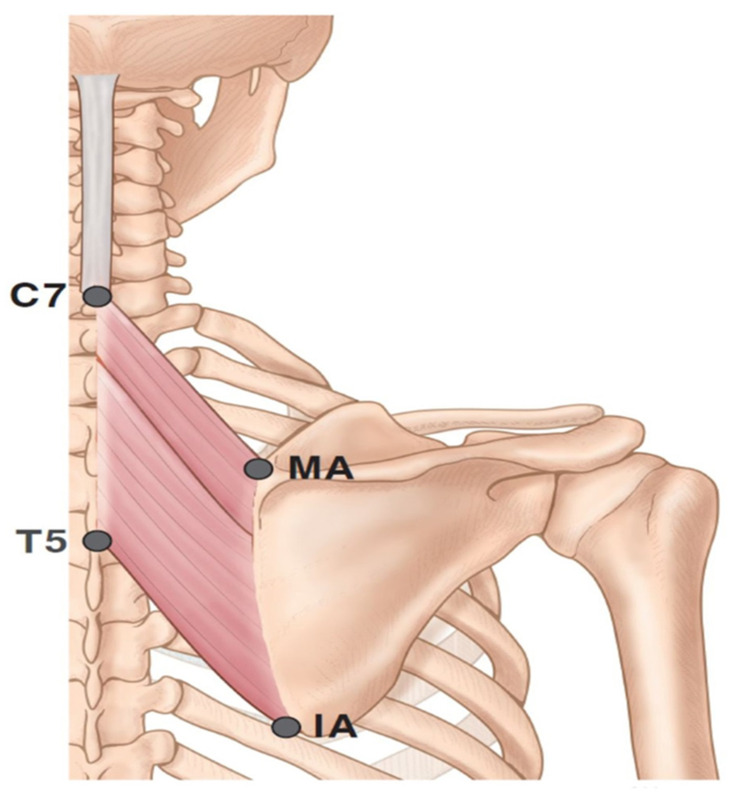
Specimens were harvested from the medial angle (MA) and inferior angle (IA) to the tips of the spinous processes of C7 and T5. The figure presents the left side of the rhomboid muscles.

Prior to dissection, the rhomboid major and minor muscles were aligned in their anatomical positions. The arborizing patterns of the muscles were traced according to four landmarks: the medial and inferior angles of the scapula and spinous processes of C7 and T5 (Figure 3). Then, we carefully recorded the nerve entry point where the nerves pierce into the muscle belly. The intramuscular neural distribution was traced with microscopic dissection before the staining procedure.

The rhomboid major and minor muscles underwent Sihler staining, as modified by Liem and Douwe van Willigen [25].

**Figure 4:**
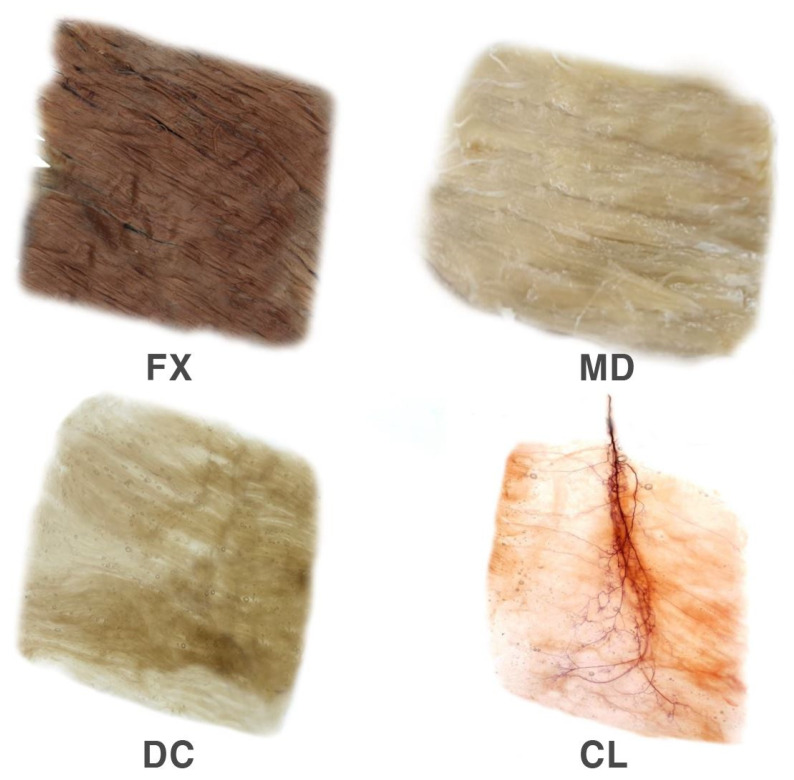
The rhomboid major muscle underwent modified Sihler’s method. The method consists of stages of fixation (FX), maceration and depigmentation (MD), decalcification (DC), staining, and clearing (CL).

This method involves multiple steps to obtain a visual representation of the intramuscular nerve arborization pattern (Figure 4).

Following Sihler staining, the rhomboid muscles were divided into five sections by an oblique line from the tips of the spinous processes (C7−T5) and three vertical lines (medial, middle, and lateral), which were equal in length.

### Modified Sihler Staining

In the fixation stage, the harvested muscles underwent one month of fixation with 10% un-neutralized formalin solution. The formalin solution was changed whenever it became opaque.

In the maceration and depigmentation stage, the fixated specimens were washed in running water for 1 h. Then, they were placed in 3% aqueous potassium hydroxide mixed with hydrogen peroxide solution for four weeks.

In the decalcification stage, the macerated specimens were placed in Sihler I solution, a compound of aqueous chloral hydrate, glycerin, and glacial acetic acid.

In the staining stage, as soon as the specimens were decalcified, they were stained in Sihler II solution, a compound of aqueous chloral hydrate, glycerin, and acetic acid, for three to four weeks.

In the destaining stage, the stained specimens were placed back in Sihler I solution to destain the muscle fibers. This procedure took 1 h.

In the neutralization stage, the destained specimens were neutralized in running water for 35 min and then immersed in a solution of 0.05% lithium carbonate for 1 h.

In the clearing stage, the neutralized specimens were cleared by increasing the concentrations of glycerin over a period of four days. The concentration of glycerin was increased in 20% increments to 100%.
